# Mitochondrial Aminoacyl-tRNA Synthetase and Disease: The Yeast Contribution for Functional Analysis of Novel Variants

**DOI:** 10.3390/ijms22094524

**Published:** 2021-04-26

**Authors:** Sonia Figuccia, Andrea Degiorgi, Camilla Ceccatelli Berti, Enrico Baruffini, Cristina Dallabona, Paola Goffrini

**Affiliations:** Department of Chemistry, Life Sciences and Environmental Sustainability, University of Parma, Parco Area delle Scienze 11/A, 43124 Parma, Italy; sonia.figuccia@unipr.it (S.F.); andrea.degiorgi@unipr.it (A.D.); camilla.ceccatelliberti@unipr.it (C.C.B.); enrico.baruffini@unipr.it (E.B.)

**Keywords:** mitochondrial aminoacyl-tRNA synthetases, yeast model, novel variants, mitochondrial diseases

## Abstract

In most eukaryotes, mitochondrial protein synthesis is essential for oxidative phosphorylation (OXPHOS) as some subunits of the respiratory chain complexes are encoded by the mitochondrial DNA (mtDNA). Mutations affecting the mitochondrial translation apparatus have been identified as a major cause of mitochondrial diseases. These mutations include either heteroplasmic mtDNA mutations in genes encoding for the mitochondrial rRNA (mtrRNA) and tRNAs (mttRNAs) or mutations in nuclear genes encoding ribosomal proteins, initiation, elongation and termination factors, tRNA-modifying enzymes, and aminoacyl-tRNA synthetases (mtARSs). Aminoacyl-tRNA synthetases (ARSs) catalyze the attachment of specific amino acids to their cognate tRNAs. Differently from most mttRNAs, which are encoded by mitochondrial genome, mtARSs are encoded by nuclear genes and then imported into the mitochondria after translation in the cytosol. Due to the extensive use of next-generation sequencing (NGS), an increasing number of mt*ARSs* variants associated with large clinical heterogeneity have been identified in recent years. Being most of these variants private or sporadic, it is crucial to assess their causative role in the disease by functional analysis in model systems. This review will focus on the contributions of the yeast *Saccharomyces cerevisiae* in the functional validation of mutations found in mt*ARS*s genes associated with human disorders.

## 1. Introduction

In most eukaryotic cells, mitochondria, where the tricarboxylic acid (TCA) cycle and oxidative phosphorylation (OXPHOS) occur, are responsible for most of the energy supply, due to their role in ATP synthesis [1]. Mitochondrial function depends on the expression of all the genes present on the mitochondrial genome and on the expression of more than a thousand of nuclear genes encoding for proteins that are imported into the organelle after translation by cytosolic ribosomes [2]. In the lower eukaryote *Saccharomyces cerevisiae*, mitochondrial DNA (mtDNA) is about 68 to 86 Kbp in size and represents on average 15% of the total cellular DNA content. It encodes eight major mitochondrial proteins that are all essential for OXPHOS: cytochrome b, a subunit of complex III; Cox1, Cox2, and Cox3, subunits of complex IV; Atp6, Atp8, and Atp9, subunits of the ATP synthase; the ribosomal protein of the small subunit Var1. Additionally, it also encodes for most of the mitochondrial tRNAs (mttRNAs) and 15S and 21S rRNAs (mtrRNAs), necessary for mitochondrial protein synthesis [3,4]. Besides tRNAs, rRNAs, and Var1, mitochondrial translation requires several other proteins, among which ribosomal proteins, initiation, elongation and termination factors, tRNA-modifying enzymes and aminoacyl-tRNA synthetases (mtARSs), which are all encoded by nuclear genes. 

ARSs ensure the proper attachment of each amino acids to its cognate tRNA [5] and are thus essential for protein synthesis. Aminoacylation of tRNA by ARSs is a two-step reaction. At first, adenosine triphosphate (ATP) reacts with the amino acid to form an aminoacyl-adenylate; in the second reaction, the amino acid is ligated to the 3′-end of tRNA forming the aminoacyl-tRNA [6]. During the amino acid activation step, misloading with a wrong amino acid can occur, especially with amino acid with similar side chains. These errors are corrected by a domain of the enzyme which is specifically designed for hydrolytic editing, to prevent mistranslation and assure the quality control of protein synthesis [7].

Although ARSs share the same mechanisms of catalysis, they are divided into two different classes (I and II) based on the active site topologies [8]. Structural studies have shown that the catalytic domain of class I contains the Rossmann dinucleotide-binding domain, whereas this fold is not present in the active site of class II enzymes, which contain a novel antiparallel β-fold. Due to the difference in the structure of the active site, class I enzymes bind ATP in an extended conformation, while those of class II binds ATP in a folded conformation [5]. Moreover, while ARSs belonging to class I approach their tRNAs via the minor groove side of the acceptor arm, class II ARSs interact with the opposite minor groove side. Consequently, attachment of the amino acid to the tRNA occurs at the 2′-OH or the 3′-OH group of the terminal adenosine, respectively [9].

In vertebrates, cytoplasmic and mitochondrial ARSs are encoded in most cases by two different genes, albeit with some exceptions as discussed in this review. Analysis of the literature shows that each of the 19 human genes encoding mitochondrial ARSs (mtARS) have been associated with a wide spectrum of human disorders [10] highlighting their importance for human health [11,12]. Although defects in all mtARSs are expected to affect mitochondrial protein synthesis, tissue-specific phenotypes have been found associated with mutations in different mtARSs [10,13]. In particular mutations in eight mtARSs generally lead to encephalopathies (mtArgRS, mtAsnRS, mtCysRS, mtIleRS, mtPheRS, mtProRS, mtThrRS, mtValRS), four to leukodystrophies (mtAlaRS, mtAspRS, mtGluRS, mtMetRS), and two to Perrault syndrome (mtHisRS, mtLeuRS). In addition, mutations in three ARSs provoke cardiomyopathies (mtAlaRS, GlyRS, LysRS), while mutations in mtTyrRS and in mtSerRS cause the MLASA syndrome and the HUPRA syndrome, respectively. Mutations also result in isolated pathological conditions such as hearing loss or deafness (mtMetRS, mtAsnRS), and intellectual disability (mt-ArgRS, mt-TrpRS) [14]. Moreover, different clinical presentations have been described in patients harboring mutations in the same gene [15], albeit the reasons for this diverse spectrum of clinical presentations are far from being understood. The total number of missense and nonsense mutations affecting the coding sequence of mt*ARS* genes is rapidly increasing. To date, 322 mutations have been found involving 311 different positions (http://misynpat.org/misynpat/) (accessed on 22 February 2021). With the exception of genes encoding glycyl- and lysyl-tRNA synthetase, for which dominant mutations have been described, all the mutations found so far in mt*ARS* genes showed a recessive inheritance. Patients are either homozygotes, 73 out of the 394 described, or compound heterozygotes.

The generation and characterization of appropriate cellular and animal models of mitochondrial diseases could help the investigation of the functional consequences of missense variants on mitochondrial metabolism, increasing our understanding on the mechanisms associated of these pathologies. In addition, these models would be valuable for searching molecules able to rescue the phenotype associated with such mutations. As recently reviewed, the unicellular and simple eukaryote *Saccharomyces cerevisiae* possesses several characteristics that have contributed to its success in increasing our knowledge on mitochondrial biogenesis and regulation such as the excellent annotation of its genome, the ease of genetic manipulation and the conservation of mitochondrial function. In the last years, studies performed in yeast have provided insights into the function of several human disease genes, particularly of those involved in mitochondrial pathologies, thanks to the yeast ability to survive without a functional respiratory chain, provided that a fermentable carbon source is made available [16,17,18,19]. 

Being mtARSs well conserved through evolution, it has been possible to exploit the yeast model to assess the pathogenic significance of novel private or sporadic variants in mt*ARS*s found by next generation sequencing (NGS). 

In this paper we will review the yeast models and the approaches that have been used for validation experiments of mutations in mt*ARS* genes. The limits of this model will be also discussed.

## 2. Functional Studies of mt*ARS* Genes Mutations in Yeast 

Thanks to extensive use of NGS, an increasing number of variants have been identified in mt*ARS* genes in recent years. In many cases patients are compound heterozygous, in which a predictably severe aminoacidic change is often combined with a predictably milder mutation [11]. Therefore, it becomes essential to assess their causative role in the disease and to evaluate the contribution of each mutation to the pathological phenotype. Functional analyses could be performed in fibroblasts from mtARS-mutant patients, but they often did not show any easily measurable readout [11]. Conversely, mutations in genes encoding for mitochondrial functions result often in a clear and distinguishable respiratory phenotype in *S. cerevisiae* that, for this reason, has been extensively used to study the consequences of potential pathological human mutations.

As recently reviewed [19], to perform validation in yeast, heterologous, homologous or chimeric complementation approach can be used depending whether or not the human cDNA can replace the yeast gene. Each of these strategies have strengths and weaknesses, and the choice depends on many factors. In all cases the wild type and mutant alleles, constructed by site specific mutagenesis, are introduced in a strain deleted of the corresponding orthologous gene and a complementation assay is first performed. 

In most cases two dedicated genes, one coding for the mitochondrial and another for the cytoplasmic isoforms, are present. However, there are some cases in which a single gene encodes for the two isoforms. Based on this feature we can distinguish four cases: (i) both humans and yeast possess a dedicated gene for each isoform (two to two); (ii) human has two distinct genes for each isoform, while in yeast a unique gene encodes for both of them (two to one); (iii) both in humans and yeast a unique gene encodes for both isoforms (one to one); (iv) in humans a unique gene encodes for both isoforms while yeast has two distinct genes (one to two) (Table 1). 

Moreover, (v) cases exist in which some genes encode for ARSs which aminoacylate tRNAs that, due to the different mitochondrial genetic code, have different specificity in humans and yeast. Finally, (vi) mt-tRNA^Gln^ is synthetized through a transamidation reaction both in humans and in yeast.

Based on the above considerations, different approaches must be used in order to validate the human mutations and minimize the chances of flaws in the results. Some examples of the different cases (Table 2) are reported in the following paragraphs together with the different functional analyses that have been performed in the yeast model. In addition, Supplementary Table S1 provides further details such as the list of all the human variants in the different mt*ARS* genes modelled in yeast, the patients’ clinical phenotype, the approach used for complementation and the associated yeast phenotypes. 

### 2.1. Two Genes in Human-Two Genes in Yeast 

Nine human genes encode for mtARS which also in yeast are encoded by a dedicated gene (Table 2). Information on the capability of the human cDNA to complement the corresponding yeast null mutant do not exist, except for *IARS2* that can partially complement the *ism1*Δ [40] but for which no disease associated variants have been modelled in yeast. 

Yeast models were used for studying mutations in *YARS2*, *DARS2*, and *WARS2* (Table 2). In all these cases, the effects of alleged mutations have been studied introducing the substitutions identified in patients in the corresponding positions of the orthologous gene: *MSY1*, *MSD1*, and *MSW1* respectively. The use of the yeast orthologous gene is based on the general assumption that if an amino acid is conserved between yeast and human ARS or lies in a highly conserved stretch, the substitution of that amino acid in the former protein can predict the effect of the mutation in the latter one. 

As a yeast model paradigm for this class of genes, we report the study of mutations in *YARS2*, encoding for the mitochondrial tyrosyl-tRNA synthetase. Mutations in this gene are associated with phenotypic heterogeneity ranging from infantile-onset and often fatal myopathy, lactic acidosis and sideroblastic anemia type 2 (MLASA2) syndrome to later adolescent-onset, slowly progressive myopathy or congenital sideroblastic anemia [22,23,24,41,42,43,44,45,46].

Since deletion of *MSY1*, as well as deletion of most mt*ARS*, leads to an irreversible loss of mitochondrial DNA [47], plasmid shuffling has been used to preserve mitochondrial functions and to validate five alleged pathological missense mutations identified in patients. Disruption of the *MSY1* gene has been performed in a haploid strain in the presence of the plasmid harboring a *URA3* marker and containing the wild type allele. This strain was then transformed with the mutant alleles, cloned under its natural promoter on a centromeric vector containing a different selection marker, and the plasmid containing the wild type gene was lost on 5-fluoroorotic acid (5-FOA)-containing medium, as previously described [19].

When the identified variants affected amino acids, which are conserved between human and yeast proteins, the mutation has been directly introduced in the corresponding amino acidic position; on the contrary, when variants affected amino acids which are not conserved, but present within conserved stretches, it has been necessary to construct also the so-called “humanized” alleles. In these cases, the amino acid of the wild type Msy1 was replaced with the amino acid present in the human wild type *YARS2*. As an example, these mutations are reported in Supplementary Figure S1. As the *msy1* humanized alleles behaved as the wild type allele, in being able to complement the oxidative growth defect of the *msy1*∆ strain, the wild type Msy1 amino acid was replaced with that found in patients. The effects of the novel missense variants were evaluated by comparing mitochondrial phenotypes such as oxidative growth phenotype, mitochondrial cytochromes content and oxygen consumption rate (OCR) between the strain carrying the mutant allele and the strain carrying the wild type and/or the humanized one. For example, a dramatic reduction of cytochromes b and aa3 was observed in the *msy1*Δ strain transformed with the *msy1*^D333E^ mutant allele, and the OCR was concordant with the cytochrome profiles. For all the *YARS2*/*MSY1* mutations studied, phenotypic analyses showed an OXPHOS defect, thus confirming pathogenicity of the mutant alleles. Given the clinical heterogeneity of this disorder, predicting disease severity in yeast could be challenging. Interestingly at this regard, the severity of the defects of the *MSY1* missense mutations modelled in yeast reflected the clinical severity of the patients carrying the corresponding mutations in *YARS2* gene [22].

Very recently, following the same strategy used for *YARS2*, several missense variants in *DARS2* gene were modeled in yeast, using the *DARS2* orthologous gene *MSD1*. *DARS2* mutations were found in patients affected by Leukoencephalopathy with brainstem and spinal cord involvement and lactate elevation (LBSL). Additionally, in this case, the functional consequences on mitochondrial metabolism associated with *DARS2* variants were in most cases in agreement with patient’s clinical phenotype [20].

### 2.2. Two Genes in Human-One Gene in Yeast 

In human, four mtARS, alanyl-tRNA synthetase, cysteinyl-tRNA synthetase, histidyl-tRNA synthetase and valyl-tRNA synthetase, are encoded by a dedicate gene, while in yeast are encoded by a unique gene, which encodes for the cytARS too (Table 2). In all these cases, the corresponding deletant strain is inviable (https://www.yeastgenome.org/) (accessed on 26 April 2021). 

No yeast model has been created so far regarding *CARS2*/*CRS1*, whereas mutations in the other three genes were modelled in yeast with different approaches (Table 2).

In yeast, the gene encoding the histidyl-tRNA synthetase (*HTS1*) has two in-frame translation start sites at the 5′ of the gene. The upstream ATG (−60) is the translation start codon for the mitochondrial isoform, whereas the downstream ATG (+1) is the translation start site for the cytoplasmic one [48,49]. In fact, removal of the first ATG by site direct mutagenesis results in a respiratory deficient phenotype, without affecting neither the level of the cytoplasmic histidyl-tRNA synthetase nor the viability. On the contrary, mutations in the second ATG lead to lethality [48]. Mutations in *HARS2* are associated to the Perrault syndrome, a disorder characterized by ovarian dysgenesis and sensorineural hearing loss [30,50,51,52]. To test the pathogenicity of alleged pathological mutations in *HARS2*, the ability of the corresponding mutations introduced into *HTS1* to complement the lethality of the deletion has been evaluated. In particular, it was shown that the mutant alleles *hts1*^L198V^ and *hts1*^V381L^ were able to fully or partially rescue, respectively, the lethality of the *hts1*Δ mutant. This suggests that the V381L mutation is more severe, whereas L198V may provide a nearly wild-type activity [30]. A similar complementation assays approach was recently used to assess the impact of new mutations in *AARS2* identified in patients presenting ataxia without leukoencephalopathy. The yeast results showed that the F102del mutation in *ALA1* (human F131del) led to loss of function, whereas the *ala1*^V306M^ (human I328M) is a hypomorphic allele [29]. It must be underlined that the approach used in these studies suffers the limit that the effects on cytoplasmic aminoacylation and on cytoplasmic protein synthesis rather than the effects on their mitochondrial counterparts are mainly evaluated, resulting in potentially flawed results.

A specific mitochondrial-focused method, that bypasses the previous issue, was used for *AARS2* and *VARS2* mutations validation [28,31,32]. Indeed, to study only the consequences of mutations on mitochondrial metabolism and avoid that some of the observed effects could be due to defects in cytoplasmic protein synthesis, it is necessary to construct a model where the wild type isoform is present in the cytoplasm only, whereas the mutant isoform is present in the mitochondrion. 

Regarding *ALA1*, it encodes both cytoplasmic and mitochondrial isoforms through alternative use of in-frame ACG triplets and a downstream ATG triplet. The mitochondrial isoform is synthetized from two adjacent in-frame non-canonical ACG (−25, −24) start codons which are upstream of the ATG (+1) start codon of its cytosolic counterpart [53]. Subsequently, the mitochondrial isoform is targeted to the mitochondria and is cleaved giving rise to the mature mitochondrial isoform [54]. In order to study the effect of a mutation on mitochondrial functionality only, at first a gene coding only for the cytosolic enzyme was produced. For this purpose, *ALA1* was mutagenized introducing a stop codon between the ACG (−25) and the ATG (+1) [28]. Since the strain with only the cytosolic function is viable but respiratory deficient, to obtain the appropriate yeast model the plasmid carrying the “cytoplasmic” allele was introduced together with the alleged pathological allele into the null strain carrying the wild type allele. Then the wild type gene was removed through plasmid shuffling.

Mutations in *AARS2* have been described associated to different disease phenotypes: infantile-onset cardiomyopathy [55,56,57,58], childhood/late adolescence/adult-onset leukoencephalopathies [59,60,61,62,63,64,65] sometimes associated to ovarian failure [28,66,67] or behavioral variant frontotemporal dementia [68], and newborn onset lethal primary pulmonary hypoplasia [69]. Moreover, mutations in *AARS2* are also associated with abnormalities in white matter [28,70], as found in LBSL disease caused by mutations in *DARS2* gene, underlying a connection between diseases produced by defects in different mt*ARS*s. 

As no biochemical readout was detected in patient cells, mutations found in *AARS2* patients were modelled in *S. cerevisiae*. In these cases, yeast gave information about the impact of mutations on the residual activity of OXPHOS complexes. For example, for the L125R and F22C mutations corresponding respectively to human L155R and F50C mutations associated with both cardiomyopathy and leukoencephalopathy, a decrease of both oxidative growth and oxygen consumption rate (OCR) was observed. To assess if the above-mentioned defects were due to an impairment of OXPHOS complexes activity caused by a defective mitochondrial protein synthesis, the activity of complex III and complex IV, the cytochrome spectra profiles and the in vivo mitochondrial protein synthesis were evaluated. Strain expressing *ala1*^L125R^ lacked complex III and complex IV activities, while strain expressing *ala1*^F22C^ showed a strong activity decrease with major impact on CIV. Cytochromes content was also decreased, as demonstrated by measuring the levels of cytochrome c, b, and aa3 spectrophotometrically. *In vivo* mitochondrial protein synthesis was performed incubating cells with cycloheximide, a cytosolic translation inhibitor, and then with [^35^S]-methionine for the time necessary to incorporate amino acids into the nascent mitochondrial proteins; total proteins were extracted, separated with SDS-PAGE, transferred to a nitrocellulose membrane and exposed to an autoradiographic film. Mitochondrial protein synthesis was absent in strain expressing *ala1*^L125R^ and decreased in strain expressing *ala1*^F22C^.

To evaluate whether the decrease or the absence of the mitochondrial protein synthesis was due to reduced levels of aminoacylated mt-tRNA^Ala^, the ability to charge this tRNA was also assessed. The analysis is based on total RNA extraction from a mitochondrial enriched fraction in acidic conditions, in order to maintain the binding between the tRNAs and their cognate amino acids. Acidic RNA extracts were separated in a partially denaturing polyacrylamide gel, electroblotted and subjected to a Northern blot analysis using as a probe a 5′-[^32^P]-labelled oligo complementary to the mt-tRNA^Ala^. The analysis revealed that the L125R substitution inhibited completely the charging of the mt-tRNA^Ala^, whereas the F22C mutation determined a partial reduction in tRNA^Ala^ loading, thus explaining the protein synthesis defects [28].

Furthermore, the F22C mutation was associated in yeast to a thermosensitive phenotype, while the L125R substitution led to a very severe defect, comparable to the absence of the gene. This suggests that the human F50C mutant protein maintains residual activity, in agreement with the slow disease progression but in contrast with the rapidly fatal outcome of the patients with cardiomyopathy [28].

Overall, the results obtained in yeast pointed out that the molecular mechanism underlying leukoencephalopathy is the charging defect. This in turn leads to the reduction of protein synthesis, a reduction of respiratory complexes activity and, finally, to OXPHOS defect. Instead, another mechanism was proposed for cardiomyopathy, namely mistranslation [28]. In fact, beside aminoacylation activity, *AARS2*/*ALA1* also possesses an editing domain responsible of removing misloaded serine or glycine [71]. A mutation impairing editing activity would determine an increase of misloaded tRNA and consequently an increased production of non-functional proteins. In this case it would be expected to observe normal mitochondrial-encoded protein levels but a significative OXPHOS defect.

The structural organization of *VAS1*, encoding the valyl-tRNA synthetase, is similar to that of *HTS1* and *ALA1*. The gene presents two in-frame start codons in the 5′ of the ORF sequence, with an upstream ATG (−46) and a downstream ATG (+1). It was show by site-directed mutagenesis that these two ATG codons encode, respectively, the initiating methionine for the mitochondrial and cytoplasmic isoforms. In fact, the disruption of the ATG (−46) codon results in a mitochondrial defect, whereas the disruption of ATG (+1) leads to lethality [72]. To model the impact of the *VARS2* mutations, only on the mitochondrial activity, the allele encoding the cytoplasmic (but not the mitochondrial) isoform of *VAS1* was generated through mutagenesis of start codon for the mitochondrial isoform which was changed to the codon for alanine GCG [31,72].

The mitochondrial *VAS1* isoform was mutagenized in order to study the effects of three mutations, one of which, T367I, was associated to mitochondrial encephalomyopathy when in homozygosis, and two, T647M and R773Q, were associated to failure to thrive and pulmonary hypertension when in compound heterozygosis [31,32]. All the equivalent mutations introduced in *VAS1* (T380I, T662M and R788Q) were associated to a decrease in the oxidative growth and in the respiratory activity, thus confirming the pathogenicity of these variants. Intriguingly, mutation T662M showed a cryosensitive phenotype, suggesting defects in the catalytic activity at low temperatures. On the contrary, the mutation R788Q resulted in a thermosensitive phenotype caused, at least in part, by protein instability, as shown by measurement of the protein levels. In addition, the oxidative growth phenotype defect of the *vas1*^T380I^ mutant strain, but not of the other strains, was completely rescued by adding valine to the medium, suggesting that increasing the substrate levels could improve the pathological phenotype.

Since the case of patient carrying the two mutations T647M and R773Q in compound heterozygosis was sporadic and the mutations have been found through the sequencing of a panel of mitochondrial genes associated to diseases, yeast has been used also to confirm that these two mutations were recessive and contribute both to the phenotype. Indeed, it is possible to easily evaluate in yeast whether a mutation is recessive or dominant and to study the effects of two mutations in compound heterozygosis [19]. The behavior of the mutation can be determined by comparing the phenotype of a heterozygous strain with that of the corresponding homozygous wild type strain. The effects of two mutations in compound can be determined comparing the phenotype of the double heterozygous strain, i.e., harboring both mutations, with that of the single heterozygous strains, i.e., harboring a single mutation in compound with the wild type allele. Both mutations in *VAS1* were recessive, as demonstrated by the phenotype of both the *VAS1*/*vas1*^T662M^ and the *VAS1*/*vas1*^R788Q^ heterozygous strains, which was similar to that of the *VAS1*/*VAS1* homozygous strain. However, in the *vas1*^T622M^/*vas1*^R788Q^ double heterozygous strain, the phenotype was compromised compared to the *VAS1*/*VAS1* homozygous strains, indicating that the pathology is indeed caused by the presence of both these mutations in compound heterozygosis [32].

### 2.3. One Gene in Human-One Gene in Yeast

The human gene *GARS* encoding glycyl-tRNA synthetase (GlyRS), [73,74] represents the only example in which both cytosolic and mitochondrial isoforms are encoded by a unique gene in humans as well as in yeast (Table 2). In humans the two isoforms are synthetized by two *GARS* start codons and differ for the *N*-terminal mitochondrial targeting sequence (MTS) of 54 amino acids [75]. The *N*-terminal WHEP-TRS domain (62–122 amino acids residue), the catalytic one (124–608) and the *C*-terminal anticodon-binding one (602–726) are the same in both isoforms [75,76]. It would be expected that mutations compromising *GARS* function affect both the cytoplasmic and mitochondrial protein synthesis; however, considering that cognate mt-tRNAs and cyt-tRNAs are different and that the concentrations of other substrates could be different in cytoplasm and mitochondria, it is possible that some mutations affect more the cytoplasmic protein synthesis than the mitochondrial one, or vice versa. In agreement with this hypothesis, the mutations identified so far in *GARS* are responsible for Charcot-Marie-Tooth disease type 2D or distal hereditary motor neuronopathy type VA, with no apparent mitochondrial defect [11]. Yeast possesses two paralogous genes encoding for the glycyl-tRNA synthetase, *GRS1*, expressed constitutively and whose deletion is lethal, and *GRS2*, expressed only in stressing conditions [77,78]. The two isoforms encoded by *GRS1* differ for the first 12 amino acids that are essential for the mitochondrial activity but not for the cytoplasmic one; this was demonstrated by the observation that the strain bearing the deletion of codons 2–12 and the insertion of a new ATG codon at position 13 is able to grow on glucose but not on oxidative carbon sources [77]. In order to model *GARS* mutations in yeast, it is possible to introduce the corresponding mutations in *GRS1* to study the effects on protein synthesis in both compartments. This is the case of two *grs1* alleged pathological alleles for which the ability of enabling cell viability for *grs1*Δ strain in media containing fermentable or respiratory carbon sources has been tested [25,79]. Moreover, it is known that a form of *GARS*, in which both MTS and WHEP-TRS domains are truncated (MTSΔWHEPΔ), is able to complement the lethality due to the deletion of *GRS1* [80,81]. This heterologous complementation approach allowed to demonstrate that I334N and G327R *GARS* variants do not support the growth of yeast, indicating a severe impairment of protein function [26,27]. 

Additionally, in this case, yeast can offer the possibility to evaluate independently the pathogenic effects of mutations in *GARS* on mitochondrial and cytoplasmic protein synthesis thanks to the possibility of constructing proper yeast models expressing a mutant isoform that localizes only in the mitochondria or only in the cytoplasm, as reported above for *ALA1* and *VAS1*.

### 2.4. One Gene in Human-Two Genes in Yeast

*KARS* is the only example of *ARS*s genes for which the cytosolic and mitochondrial isoforms are encoded by a unique gene in human and by two different genes in yeast, *KRS1* and *MSK1*, respectively (Table 2). An alternative splicing of *KARS* generated the two isoforms of lysyl-tRNA synthetase. The cytoplasmic isoform (cytLysRS) is generated by pre-mRNA splicing of exons 1–3; the mitochondrial isoform (mtLysRS) is generated by pre-mRNA splicing that retains exon 2, which encodes for the mitochondrial signal peptide [82,83]. Mutations in *KARS* are associated with a wide spectrum of clinical manifestations, including sensorineural hearing loss [84], optic neuropathy [85], peripheral neuropathy [86], hypertrophic cardiomyopathy with lactic acidosis, and complex I and IV deficiency [87,88]. 

In *S. cerevisiae*, *KRS1* encodes the cytLystRS whose deletion is lethal, whereas *MSK1* encodes the mtLysRS whose loss results in respiratory deficiency and instability of mitochondrial DNA (mtDNA) [89]. It is known that human cDNA encoding for mtLysRS, under the control of an appropriate yeast promoter and cloned in a multicopy vector, is able to complement the yeast null strain *msk1*Δ [90]; moreover, it has been recently demonstrated that the multicopy expression of the cytLysRS [35] or the expression of a truncated form of the cytLysRS lacking *N*-terminal sequence Met1-Ser70 [34] are able to complement the *krs1*Δ null strain. Taking advantage of the heterologous complementation approach, it has been possible to investigate separately the effects of identified variants, by introducing the mutations in each isoform and transforming separately the strains deleted for *MSK1* or *KRS1*. The mitochondrial phenotypes (oxidative growth, OCR, and mitochondrial protein synthesis) of *msk1*Δ strain harboring mtLysRS mutant alleles, or the ability of cytLysRS mutant alleles to complement the *krs1*Δ lethal phenotype have been evaluated [35]. Results demonstrated a detrimental role of the mutations both on the mitochondrial phenotypes and on the viability, indicating that each mutation affects both isoforms. However, the R205C, H402Y, and N591I variants decreased or abolished the oxidative growth of mtLysRS mutant strains, whereas they induced only a moderate growth defect on cytLysRS mutant strains. On the contrary, the P499L and R348C variants did not affect the oxidative growth of mtLysRS mutant strains, but severely affected the viability of cytLysRS mutant strains. This suggested that for some mutations a detrimental effect in one of the two compartments is predominant. In addition, growth defects of cytLysRS mutant strains harboring mutations P533R and P585C, but not of the corresponding mtLysRS mutant strains, are partially rescued by supplementing the medium with lysine, suggesting the catalytic defects are due to a decreased affinity for the substrate. 

### 2.5. Specific Cases

*RARS2* and *LARS2* encode for the mitochondrial arginyl-tRNA synthetase and the mitochondrial leucyl-tRNA synthetase; these are encoded also in yeast by a dedicated gene: *MSR1* and *NAM2*, respectively. Although the yeast enzymes catalyze the amino acid adenylation reaction of their human counterpart, they differ in their cognate tRNAs due to differences in the mitochondrial genetic code between human and yeast.

As schematized in Table 3, in humans RARS2 charges only mt-tRNA(UCG), which binds canonical CGN codons (where N indicates any nitrogen base among A, C, G, and U); on the contrary, Msr1 charges both the mt-tRNA(UCU), which in yeast mitochondria binds AGR codons (where R indicates a purine) that in humans mitochondria are stop codons, and the mt-tRNA(ACG), which binds the canonical CGN codons. LARS2, charges both the mt-tRNA(UAG), which binds canonical CUN codons, and the mt-tRNA(UAA), which binds canonical UUR codons. On the contrary, yeast Nam2, charges only the mt-tRNA(UAA), which binds canonical UUR codons, since CUN codons encode for threonine. 

It is important to note that in these two cases the yeast model might not exactly mimic the human condition since the aminoacyl-tRNA synthetase may differ in the anticodon binding domain sequence due to the loading of a different tRNA in humans. For this reason, yeast might fail in modeling mutations that lie in the anticodon binding domain. Therefore, it possible to use the yeast for studying the consequences of pathological mutations associated to *RARS2* and *LARS2*, provided that amino acids under analysis are not predicted to be involved in the binding of those tRNA whose anticodon has a different specificity. It is possible that the two models described below have not always managed to validate the pathological role of an amino acid substitution for this reason.

*MSR1* was used to model human *RARS2* recessive mutations W241R, R245Q, and R469H, all associated to pontocerebellar hypoplasia type 6: while the pathogenicity of the last two mutations was demonstrated in yeast, the first mutation was not associated to a detrimental phenotype, suggesting that this yeast model could not be enough sensitive, especially if the allele is hypomorphic [37]. Heterologous expression of *LARS2* in a *nam2Δ* strain was used to model recessive mutations I360FfsTer15, T522A, and T629M, which are associated to premature ovarian failure and hearing loss. The pathogenicity for the first two mutations was demonstrated in the yeast model, but not for the latter, suggesting again a low sensitivity for some mutant alleles [36].

A similar but not identical case is represented by *TARS2* which encodes the human mitochondrial threonyl-tRNA synthetase. In yeast this activity is encoded by two genes, *THS1* and *MST1*. The first gene encodes both the cytoplasmic isoform and, based on high throughput analysis, for an isoform that functions also in mitochondria; this mitochondrial-active isoform could charge only the canonical mt-tRNA(UGU), which binds ACN codons. The *MST1* gene encodes for the main mitochondrial isoform, which charges both the canonical mt-tRNA(UGU) and the non-canonical mt-tRNA(UAG), which binds CUN codons (Table 3). In contrast to most eukaryotic ARSs, Mst1 lacks the *N*-terminal post-transfer editing domain and, for this reason, it is considered an error prone synthetase. In fact, the lack of a post-transfer editing domain prevents to hydrolyze the link between tRNA^Thr^ and an eventual misloaded amino acid, especially serine [38,91,92]. Mutations in *TARS2* gene are associated in humans to fatal mitochondrial encephalomyopathy [93], which manifests itself with axial hypotonia and limb hypertonia, psychomotor delay, and high levels of blood lactate [31]. These mutations cannot be studied in *MST1* gene, since Mst1 has been demonstrated to have a different structure compared to other ThrRS [94] and to recognize acceptors tRNAs via different mechanisms [92]. These mutations cannot be even studied directly by introducing them in the equivalent position of *THS1* that presents a more similar structure; in fact, provided that Ths1 localizes also in mitochondria, it is not able to charge mt-tRNA(UAG). In this regard, it should be underlined that it was observed that human *TARS2* can partially complement the lethal effect due to the deletion of *THS1*, when *TARS2* lacking the first 19 codons is coexpressed in the cytoplasm with the human cytRNA^Thr^(AGU) [38]. However, this system failed to demonstrate *in vivo* the pathogenicity of P282L mutation, when either mutant *TARS2* or *THS1* mutated in the equivalent position were expressed. Although the mutation in *THS1* resulted in a decrease of the protein levels by 75%, no growth defects were observed; this is likely due to a 5% residual activity of the cytoplasmic ThrRS which is sufficient to fully sustain protein synthesis, making this system unsuitable for modelling hypomorphic alleles which retains more than 5% activity [91].

### 2.6. The Peculiar Case of the GatCAB Complex

Each tRNA is aminoacylated by a specific ARS, except for glutaminyl-tRNA (mt-tRNA^Gln^) that in mitochondria, as in most bacteria, is aminoacylated though an indirect two-step pathway. The first reaction is in fact generally catalyzed by the glutamyl-tRNA synthetase (GluRS), which loads glutamic acid not only on the tRNA^Glu^, but also on the tRNA^Gln^, generating in the latter case a mischarged Glu-tRNA^Gln^ [95,96]. In humans this reaction is catalyzed by the mitochondrial glutamyl-tRNA synthetase *EARS2*. Differently, in *S. cerevisiae*, during the switching from a fermentative to a respiratory metabolism, the cytosolic glutamyl-tRNA synthetase encoded by *GUS1*, is imported into mitochondria and loads only the glutamate on the mt-tRNA^Gln^ [97]. In a second reaction, the resulting mischarged Glu-tRNA^Gln^ is then converted into Gln-tRNA^Gln^ by glutamyl-tRNA^Gln^ amidotransferase (GluAdT) complex, using glutamine as amide donor, [98,99].

The human mitochondrial glutamyl-tRNA^Gln^ amidotransferase is a heterotrimeric enzyme complex, called GatCAB, that consists of the A, B, and C subunits encoded by *QRSL1*, *GATB*, *GATC* genes, respectively. GATB subunit phosphorylates the glutamyl-carboxyl group on the mischarged tRNA; this activated intermediate is then amidated by GATA subunit [98,100]. On the other hand, *GATC* encodes for a subunit with a structural function that is able to stabilize and connects the two catalytic subunits [101,102]. Mutations in all the genes that encode for the GatCAB subunits were recently associated with fatal cardiomyopathy and lactic acidosis [39,56,87], leading to the completion of the collection of mitochondrial aminoacylation defects in human diseases, which began in 2007 with the discovery of pathogenic mutations in *DARS2* [103].

In *S. cerevisiae*, the glutamyl-tRNA^Gln^ amidotransferase mitochondrial complex, called GatFAB, is composed by three subunits, encoded by the *HER2*, *PET112* and *GTF1* genes; the former two genes are orthologue of *QRSL1* and *GATB*, respectively, whereas no sequence similarity exists between human *GATC* and yeast *GTF1*. *HER2* and *PET112* have been used to study the effect of human pathological mutations in the corresponding genes by homologous complementation. 

*HER2* and *PET112* are essential for mitochondrial function; in fact, their disruption destabilize mtDNA and makes the strain respiratory deficient [104,105]. As an example of validation of mutations affecting GatCAB complex, two new allegedly pathological alleles of *QRSL1,* identified by WES in compound heterozygosis in a patient affected by severe cardiomyopathy, are reported; three substitutions, T196N, R197K and P199H, were found in the paternal gene, whereas a single substitution, A427L, was detected in the maternal gene [39]. The variants were introduced in the corresponding positions of Her2, generating the *her2*^V155N-R156K-P158H^ and *her2*^P397L^ alleles, which were introduced trough plasmid shuffling in a *her2Δ* strain. Oxidative growth and OCR were absent in the mutant strain harboring the triple mutant alleles, whereas these were only partially impaired in the mutant strain harboring the single mutant allele, indicating that the paternal allele is null whereas the maternal allele is hypomorphic; these results indicate that both variants are pathological and causative of the mitochondrial disease observed in patients.

Regarding *GATB* gene, one new missense mutation (F136L) was identified in a patient and modelled in the yeast orthologues gene *PET112* by the same strategy used for *QRSL1*/*HER2*. Interestingly, OCR reduction of the mutant strain *pet112*^F103L^ was strongly exacerbated at high temperature (37 °C) and in absence of the amide donor glutamine. These data support the pathogenic role of the P136L mutation and suggest a possible rescuing effect by the supplementation of glutamine.

## 3. Conclusions

Mitochondrial aminoacyl tRNA synthetases (mtARSs) are a group of enzymes essential for protein synthesis within the mitochondrial compartment. MtARSs are encoded by nuclear DNA and imported in the organelle after translation in the cytoplasm. To date, mutations in all the 19 mt*ARS*s genes have been identified and associated to mitochondrial diseases presenting a vast range of clinical phenotypes. Next-generation sequencing technology allowed the identifications of a huge number of variants in mt*ARSs* genes, whose pathogenicity must be carefully evaluated, especially when they are novel, sporadic or private. Moreover, patients are often compound heterozygous, and for this reason it is important to evaluate the contribution of each amino acid change to enzyme activities and, ultimately, to pathological phenotypes. To this end, several approaches can be used, and functional studies are needed for predicting pathogenicity. These analyses could be done in cultured cells such as fibroblasts; however, in most cases, this approach is hindered by the lack of an evident phenotype [13]. 

Although yeast, as a single-cell microorganism, cannot be used for studying the effect of alleged pathological mutations at the level of tissues or organs, or for understanding the heterogeneity of the phenotype which is often associated to mutations in such genes, it has been successfully used to study the consequences of mt*ARS* mutations on intracellular metabolism. In the last years, several yeast models carrying novel mt*ARS* variants have been constructed. In most cases, the ability/inability of the alleged pathological alleles to support oxidative growth and respiratory activity of the mutated strain contributed to validate the mutations and to assess their role when present in compound in patients. 

Flaws in mitochondrial aminoacylation results in defects in protein translation, which should affect the synthesis of all the complexes subunits encoded by the mtDNA and, therefore, the activity of all the respiratory complexes (except for complex II which is exclusively encoded by nuclear genes). However, if mutations in mt*ARS* genes affect only and specifically NADH:ubiquinone oxidoreductase complex, yeast might not be the best model for modelling these mutations: *S. cerevisiae* does not possess respiratory complex I and thus no genes encoding for subunits of such complex are present on the mtDNA. 

One of the most important advantages of using yeast is that it is possible to determine the consequences of each single variant in an identical genetic background. Indeed, in patients, who are genetically heterogeneous, there may be other indeterminate and unknown genetic factors that influences the severity and the progression of the disease. For example, it has been hypothesized that mtDNA haplotype background may influence phenotypic expression in the case of patients presenting *YARS2* mutations [22].

Moreover, the severity of the impairment of mitochondrial function observed in yeast often correlates with the severity of the clinical phenotype of the patients [20,22]

As mentioned previously, in most cases mtARSs are encoded by a dedicated gene; however, exceptions exist in which, both in humans and yeast, a unique gene encodes the mitochondrial and the cytoplasmic isoforms via alternative translation, or a dedicated gene for mtARSs is present in the human but not in the yeast genome or vice versa. When variants are found in these mt*ARS* genes, yeast offers the possibility to establish the impact of the variants specifically on mitochondrial function by constructing a model in which the mutant isoform is present only in the mitochondrion [28,31,32].

Since in many cases mt*ARS*s pathological mutations are not associated to a complete loss of function but maintain a partial enzymatic activity, it is possible that an increased availability of the amino acid substrate for a given mtARS could improve the catalytic efficiency of the mutant enzyme. A beneficial effect of amino acids supplementation has been observed in different mtARS in vitro models and in some yeast mutant strains (e.g *vas1*^T380I^). However, the approach did not always produce the same beneficial effect, and a correspondence between different models has not been always observed. This suggests that the response to amino acid supplementation could be gene- or mutation-specific and hardly to predict in simple models. However, if a beneficial effect deriving from the supplementation of the substrate amino acid is observed in yeast, it is certainly worth to experiment amino acid supplementation in mt*ARS*-patients. In fact, substrate enhancement, which in some cases can be obtained through dietary supplementation, is an emerging treatment for mitochondrial pathologies [106]. 

One of the substrates of each mtARS is its cognate tRNA. Although the structure of each specific tRNA is evolutionary conserved, the sequence, including that of the anticodon, can be different in yeast and humans. Besides the different codon specificity reported above, it is possible that the binding of the cognate tRNA is slightly different between the two species, and that amino acids which are critical in human for such binding are not fundamental for the binding in yeast. Indeed, it is possible that the insertion in yeast of a mutation in amino acids involved in the tRNA recognizing and binding could not reflect the detrimental effects in the corresponding position of the human mtARS, thus leading to a lack of a “pathological” phenotype. For this reason, if a detrimental effect is observed in a mutant yeast strain, as observed for most mt*ARS* mutations studied until now, it is highly probable that the mutation is also pathogenic in humans; on the contrary, if no detrimental effects are observed in yeast, it cannot be excluded that the mutation is pathogenic in humans. 

However, despite these limitations, the similarities between the yeast and the human mitochondrial translation machineries makes yeast a good model to evaluate in a quick and efficient way the effects of mutations in human genes encoding for mtARSs.

## Figures and Tables

**Table 1:** *ARS* encoding genes in human and yeast. In both organisms, a unique gene (*ARS*) or two specific genes encode for the mitochondrial and cytoplasmatic aminoacyl-tRNA synthetase isoforms, (mtARS and cytARS, respectively). The different combinations can be clustered in 4 different cases.

Cases	Human	Yeast
Two to Two	mt*ARS*, cyt*ARS*	mt*ARS*, cyt*ARS*
Two to one	mt*ARS*, cyt*ARS*	*ARS*
One to One	*ARS*	*ARS*
One to Two	*ARS*	mt*ARS*, cyt*ARS*

**Table 2:** List of human mt*ARS* genes and their corresponding yeast orthologues. For each gene, the associated human diseases with OMIM number and the publications where yeast was used to validate the pathogenic role of the variants identified in patients are reported.

Human Gene vs. Yeast Gene	Protein	Human Gene	OMIM Phenotype	Yeast Gene(s)	Yeast Model
Two to Two	mt aspartyl-tRNA synthetase	*DARS2*	#611105: leukoencephalopathy with brainstem and spinal cord involvement and lactate elevation, LBSL	*MSD1*	[20]
	mt phenylalanyl-tRNA synthetase	*FARS2*	#614946: combined oxidative phosphorylation deficiency 14; COXPD14 #617046: spastic paraplegia 77, autosomal recessive, SPG77 #203700: mitochondrial DNA depletion syndrome 4A (Alpers type); MTDPS4A	*MSF1*	no yeast model described
	mt isoleucyl-tRNA synthetase	*IARS2*	#616007: cataracts, growth hormone deficiency, sensory neuropathy, sensorineural hearing loss, and skeletal dysplasia; CAGSSS #256000: Leigh syndrome; LS	*ISM1*	no yeast model described
	mt methionyl-tRNA synthetase	*MARS2*	#611390: spastic ataxia 3, autosomal recessive; SPAX3 #616430: combined oxidative phosphorylation deficiency 25; COXPD25	*MSM1*	no yeast model described
	mt asparaginyl-tRNA synthetase	*NARS2*	#618434: deafness, autosomal recessive 94; DFNB94 #203700: mitochondrial DNA depletion syndrome 4A (Alpers type); MTDPS4A #616239: combined oxidative phosphorylation deficiency 24; COXPD24	*SLM5*	no yeast model described
	mt prolyl-tRNA synthetase	*PARS2*	#618437: developmental and epileptic encephalopathy 75; DEE75	*AIM10*	no yeast model described
	mt seryl-tRNA synthetase	*SARS2*	#613845: hyperuricemia, pulmonary hypertension, renal failure, and alkalosis; HUPRAS	*DIA4*	no yeast model described
	mt tryptophanyl-tRNA synthetase	*WARS2*	#617710: neurodevelopmental disorder, mitochondrial, with abnormal movements and lactic acidosis, with or without seizures; NEMMLAS	*MSW1*	[21]
	mt tyrosyl-tRNA synthetase	*YARS2*	#613561: myopathy, lactic acidosis, and sideroblastic anemia 2; MLASA2	*MSY1*	[22,23,24]
One to One	mt and cyt glycyl-tRNA synthetase	*GARS*	#601472: Charcot-Marie-Tooth disease, axonal, type 2D; CMTD2 #600794: neuronopathy, distal hereditary motor, type VA; HMN5A #619042: spinal muscular atrophy, infantile, James type; SMAJI	*GRS1*	[25,26,27]
Two to One	mt alanyl-tRNA synthetase	*AARS2*	#615889: leukoencephalopathy, progressive, with ovarian failure; LKENP #614096: combined oxidative phosphorylation deficiency 8; COXPD8	*ALA1*	[28,29]
	mt cysteinyl-tRNA synthetase	*CARS2*	#616672: combined oxidative phosphorylation deficiency 27; COXPD27	*CRS1*	no yeast model described
	mt histidyl-tRNA synthetase	*HARS2*	#614926: Perrault syndrome 2; PRLTS2	*HTS1*	[30]
	mt valyl-tRNA synthetase	*VARS2*	#615917: combined oxidative phosphorylation deficiency 20; COXPD20	*VAS1*	[31,32]
One to Two	mt and cyt lysyl-tRNA synthetase	*KARS*	#613641: Charcot-Marie-Tooth disease, recessive intermediate, B; CMTRIB #613916: deafness, autosomal recessive 89; DFNB89 #619196: deafness, congenital, and adult-onset progressive leukoencephalopathy; DEAPLE #619147: leukoencephalopathy, progressive, infantile-onset, with or without deafness; LEPID	*KRS1* *MSK1*	[33,34,35]
Others	mt leucyl-tRNA synthetase	*LARS2*	#615300: Perrault syndrome 4; PRLTS4 #617021: hydrops, lactic acidosis, and sideroblastic anemia; HLASA	*NAM2*	[36]
	mt arginyl-tRNA synthetase	*RARS2*	#611523: pontocerebellar hypoplasia, type 6; PCH6	*MSR1*	[37]
	mt threonyl-tRNA synteatse	*TARS2*	#615918: combined oxidative phosphorylation deficiency 21; COXPD21	*THS1* *MST1*	[38]
	mt glutamyl-tRNA and mt glutamyl-tRNA^Gln^ synthetase	*EARS2*	#614924: combined oxidative phosphorylation deficiency 12; COXPD12	MSE1/GUS1 *	no yeast model described
	GatTCAB complex	*QRSL1*	#618835: combined oxidative phosphorylation deficiency 40; COXPD40	*HER2*	[39]
		*GATB*	#618838: combined oxidative phosphorylation deficiency 41; COXPD41	*PET112*	
		*GATC*	#618839: combined oxidative phosphorylation deficiency 42; COXPD42	*GTF1*	

* In yeast, the mitochondrial glutamyl tRNA^Glu^-synthetase activity (producing mt-GlutRNA^Glu^) is catalyzed by Mse1 whereas the mitochondrial glutamyl tRNA^Gln^-synthetase activity (producing mt-GlutRNA^Gln^) is catalyzed by the cytoplasmic Gus1 protein which, during respiratory metabolism, is imported into mitochondria.

**Table 3:** Different anticodon/codon specificity for mt-tRNA in humans and in yeast. Amino acids not encoded according to the standard genetic code are highlighted in italics. N indicates any nucleotide, R a purine.

Human/Yeast mtARS	Anticodon-Codon mt Human	Anticodon-Codon mt Yeast	aa Codified in Human	aa Codified in Yeast
RARS2/Msr1	UCG-CGN	UCG-CGN	Arg	Arg
	-	UCU-AGR	*Ter*	Arg
LARS2/Nam2	UAA-UUR	UAA-UUR	Leu	Leu
	UAG-CUN	-	Leu	*Thr*
TARS2/Mst1	UGU-ACN	UGU-ACN	Thr	Thr
	-	UAG-CUN	*Leu*	Thr

## Data Availability

Not applicable.

## Supplementary Materials

The following are available online at https://www.mdpi.com/article/10.3390/ijms22094524/s1, Figure S1: Alignment of human YARS and yeast Msy1 proteins with % identity and similarity, Table S1 Human and yeast phenotype associated with mutations in ARS2.
