# Supplementary material for: Mitochondrial Aminoacyl-tRNA Synthetase and Disease: The Yeast Contribution for Functional Analysis of Novel Variants
Source: Int J Mol Sci. 2021 Apr 26;22(9):4524. doi: 10.3390/ijms22094524 (PMC8123711; doi:10.3390/ijms22094524)
Supplement: Supplementary file 1 [file ijms-22-04524-s001.zip › Supplementary Figure 1.pdf]

Supplementary Figure 1. Alignment of human YARS and yeast Msy1 proteins with % identity and similarity. Alignment was performed with Clustal Omega at https://www.ebi.ac.uk/Tools/msa/clustalo/, using default parameters. Mutated amino acids modeled in yeast through homologous complementation are highlighted in green if conserved and in yellow if present in conserved stretches, thus requiring construction of "humanized alleles".

```
hYARS2          ---------MAAPILRSFSWGRWSGTLNLSVL------LPLGL---RKAHSGAQGLLAAQ     42
yMsy1           MLELRSCSNLVNSSRRLVPLVTYSGLSAITLPKSRFYSQPSALEVQGTSDSRSDNILDEL     60
                         :.    * .    :**   :::        * .*    .:.* ::.:*

hYARS2          KARGLFKDFFPETGTKIELPELFDRG--TASFPQTIYCGFDPTADSLHVGHLLALLGLFH     100
yMsy1           KQRGLVSQ--------VSQPESFLRTKLNGNDKIKLYCGVDPTAQSLHLGNLVPLMVLLH     112
                * ***..:        :. ** * *   ...   .:***.****:***:*:*: *: *:*

hYARS2          LQRAGHNVIALVGGATARLGDPSGRTKEREALETERVRANARALRLGLEALAANH-----     155
yMsy1           FYVKGHDIVTVIGGATGKVGDPSGRKTERDVMENDIRQSNVASISQQLQRFFKNGLEYYR     172
                :   **::::::****.::******..**:.:*.:  ::*. ::   *: :  *

hYARS2          -QQLFTDGRSWGSFTVLDNSAWYQKQHLVDFLAAVGGHFRMGTLLSRQSVQLRLKSPEGM     214
yMsy1           NRCALTEDVPSGKYTPRNNFNWWKDIKMLDFLADFGRHIRVQSMLARDSISSRLQTKNGL     232
                 :  :*:.   *.:*  :*  *::. :::**** .* *:*: ::*:*:*:. **:: :*:

hYARS2          SLAEFFYQVLQAYDFYYLFQRYGCRVQLGGSDQLGNIMSGYEFINKLTG----EDVFGIT     270
yMsy1           GFNEFTYQVLQAYDFYHLYKEENVTIQVGGNDQWGNITAGIDLINRIQPIKNKGLPFGIT     292
                .: ** **********:*::. .  :*:**.** *** :* ::**::         ****

hYARS2          VPLITSTTGAKLGKSAGNAVWLNRDKTSPFELYQFFVRQPDDSVERYLKLFTFLPLPEID     330
yMsy1           VPLLTTATGEKFGKSAGNAVFIDPSINTAYDVYQFFYNTLDADVPKFLKIFTFLNSSEIK     352
                ***:*::** *:********::: . .: :::**** .  * .* ::**:****   **.

hYARS2          HIMQLHVKEPERRGPQKRLAAEVTKLVHGREGLDSAKRCTQALYHSSIDALEVMSDQELK     390
yMsy1           KIVETHIKSPSLRYGQTLLAKEVTDMLYGVGSGSDSEALSNIIFGRYDGT---LSAAKLV     409
                :*:: *:*.*. *  *. ** ***.:::*  . ..::  :: ::     .:    :*   :*

hYARS2          ELFKEAPFSEFFLDPGTSVLDTCRKANAIPDGPRGYRMITEGGVSINHQQVTNPESVLIV     450
yMsy1           DLCKKARILQYADREIDLIKLICKLVNCSV--SEARRKLSQGSVYLHHSKSKVNENISNL     467
                :* *:* : ::       :   *: .*.     .. * :::*.* ::*.: .  *.:  :

hYARS2          GQHILKNGLSLLKIGKRNFYIIKWLQL   477
yMsy1           APFLIDDRVLILRIGKQKCFIIEMR--   492
                . .::.: : :*:***:: :**:
```

Identity: 31,1%
Similarity: 66,9%
