# Supplementary material for: Mitochondrial Aminoacyl-tRNA Synthetase and Disease: The Yeast Contribution for Functional Analysis of Novel Variants
Source: Int J Mol Sci. 2021 Apr 26;22(9):4524. doi: 10.3390/ijms22094524 (PMC8123711; doi:10.3390/ijms22094524)
Supplement: Supplementary file 1 [file ijms-22-04524-s001.zip › Supplementary table 1.pdf]

Human *ARS* gene	Protein substitution modeled in yeast	Clinical phenotype associated to the substitution	Yeast model	Yeast mutant phenotype
AARS2	Phe50Cys	Leukoencephalopathy with ovarian failure. In compound heterozygosis with Arg521*	*ala1Δ* strain expressing *ALA1* mutant alleles cloned in a centromeric plasmid under its natural promoter in presence of *cytALA1* wild type allele (1)<br><br>*ala1Δ* strain expressing human *AARS* mutant alleles cloned in a multicopy plasmid under ADH promoter (2)	(1) Thermosensitive strong decrease in oxidative growth, OCR, mitochondrial protein synthesis, ability to charge tRNA, OXPHOS complexes III and IV activities and cytochromes content.
	Phe131*	Ataxia without leukoencephalopathy. In compound heterozygosis with Ile328Met		(1)(2) Unable to support yeast growth
	Leu155Arg	Cardiomyopathy. In compound heterozygosis with Arg592Trp		(1) Strong decrease in OCR and cytochromes content. Absent oxidative growth, mitochondrial protein synthesis, ability to charge tRNA and OXPHOS complexes III and IV activities
	Ile328Met	Ataxia without leukoencephalopathy. In compound heterozygosis with Phe131*		(1)(2) Similar yeast cell growth compared to wildtype
	Arg521*	Leukoencephalopathy with ovarian failure. In compound heterozygosis with Phe50Cys		(1) Strong decrease in OCR and cytochromes content. Absent oxidative growth, mitochondrial protein synthesis, ability to charge tRNA and OXPHOS complexes III and IV activities
	Arg58Gly	LBSL-group 1[1], in compound heterozygosis with Gln248*		Mild decrease in OCR
	Arg179Cys	LBSL-group 1[1], in compound heterozygosis with Ser597Gly		Strong decrease in oxidative growth and OCR, strong increase in protein levels
	Leu250Val	LBSL-group 1[1], in compound heterozygosis with Phe488Leu		Strong decrease in oxidative growth and OCR, mild decrease in protein levels

	DARS2	Ala262Val	LBSL-group 1[1], in compound heterozygosis with Tyr30*	*msd1Δ* strain expressing *MSD1* mutant alleles cloned in a centromeric plasmid under its natural promoter	Moderate decrease in oxidative growth and OCR
		Val481Met	LBSL-group 1[1], in compound heterozygosis with Gly128Arg		Thermosensitive strong decrease in oxidative growth and in OCR
		Phe484Leu	LBSL-group 1[1], in compound heterozygosis with Leu250Val		Strong decrease in oxidative growth and OCR
		Leu588Val	LBSL-group 2[2], in compound heterozygosis with Arg188* or Met1?		Strong decrease in oxidative growth and OCR, mild decrease in protein levels
		Ser597Gly	LBSL-group 1[1], in compound heterozygosis with Arg179Cys		Strong decrease in oxidative growth and OCR, strong increase in protein levels
		Lys606Met	LBSL-group 2[2], in compound heterozygosis with Arg76Serfs*5		Mild decrease in OCR, mild increase in protein levels
	GARS	Asp146Asn	Charcot-Marie-Tooth disease. Dominant mutation	*grs1Δ* strain expressing *GRS1* mutant alleles cloned in a centromeric plasmid under its natural promoter (1)<br><br>*grs1Δ* strain expressing human *GARS* MTSΔ[3]WHEPΔ[4] mutant alleles cloned in a multicopy plasmid under ADH promoter (2)	(1) Severe reduction of yeast cell growth
		Pro244Leu	Charcot-Marie-Tooth disease. Dominant mutation		(1) Severe reduction of yeast cell growth
		Ile280Phe	Charcot-Marie-Tooth disease. Dominant mutation		(1) Similar yeast cell growth compared to wildtype
		Gly327Arg	Distal motor neuropathy. Dominant mutation		(2) Unable to support yeast growth
		Ile334Asn	Infantile SMA[5]. Dominant mutation		(2) Unable to support yeast growth
GaTCAB complex	*QRSL1*	Gly133Val	Fatal cardiomyopathy and lactic acidosis. In compound heterozygosis with Tyr185*	*her2Δ* strain expressing *HER2* mutant alleles cloned in a centromeric plasmid under its natural promoter	Moderate reduction in oxidative growth and severe reduction in OCR
		Thr196Asn			Absent oxidative growth and OCR

		Arg197Lys	Fatal cardiomyopathy and lactic acidosis. These three missense mutations are found on the same allele in compound heterozygosis with Ala427Leu		
		Pro199His			
		Ala427Leu	Fatal cardiomyopathy and lactic acidosis. In compound heterozygosis with Thr196Asn, Arg197Lys and Pro199His		MIld reduction in oxidative growth and OCR
	GATB	Phe136Leu	Fatal cardiomyopathy and lactic acidosis. In compound heterozygosis with Ser194Trpfs*15	*pet112Δ* strain expressing *PET112* mutant alleles cloned in a centromeric plasmid under its natural promoter	Thermosensitive moderate reduction in oxidative growth and severe reduction in OCR
HARS2		Leu200Val	Perrault syndrome. Δ200-211 on the same allele, in compound heterozygosis with Val368Leu	*hts1Δ* strain expressing *HTS1* mutant alleles cloned in a centromeric plasmid under its natural promoter	Similar yeast cell growth compared to wildtype
		Δ200-211	Perrault syndrome. Leu200Val on the same allele, in compound heterozygosis with Val368Leu		Unable to support yeast growth
		Val368Leu	Perrault syndrome. In compound heterozygosis with Leu200Val and Δ200-211		Reduced yeast cell growth
KARS		Ala2Val	Developmental delay, intellectual disability and/or microcephaly. In compound heterozygosis with Arg108Cys	*krs1Δ* strain expressing *KRS1* mutant alleles cloned in a centromeric plasmid under its natural promoter (1)	(3) Increased yeast growth

	Ala57Pro	Developmental delay, intellectual disability and/or microcephaly. In compound heterozygosis with Pro533Arg	*msk1Δ* strain expressing human *mtKARS* mutant alleles cloned in a centromeric plasmid under the tetO-CYC1 promoter (2)<br><br>*krs1Δ* strain expressing human *cytKARS* mutant alleles cloned in a multicopy plasmid under the tetO-CYC1 promoter (3)	(2) Thermosensitive mild reduction in oxidative growth and OCR; mild reduction in mitochondrial protein synthesis<br>(3) Mild reduced yeast growth
	Arg108Cys	Developmental delay, intellectual disability and/or microcephaly. In compound heterozygosis with Ala2Val		(2) Absent oxidative growth, OCR and mitochondrial protein synthesis. Moderate severe reduction of protein levels<br>(3) Unable to support yeast growth
	Tyr173His	ARNSHI[6]. Homozygosis		(1) Mild reduced yeast growth<br>(2) Absent oxidative growth<br>(3) Moderate reduced yeast growth
	Arg205Cys	Developmental delay, intellectual disability and/or microcephaly. Homozygosis		(2) Severe decrease in oxidative growth, OCR and mitochondrial protein synthesis<br>(3) Moderate reduced yeast growth
	Leu233Val	Leukoencephalopathy. Homozygosis		(1) Mild reduced yeast growth
	Phe291Val	Developmental delay, intellectual disability and/or microcephaly. In compound heterozygosis with Pro499Leu		(2) Mild reduction in oxidative growth, severe decrease in OCR and markedly reduced mitochondrial protein synthesis<br>(3) Moderate reduced yeast growth
	Ile346Thr	Developmental delay, intellectual disability and/or microcephaly. Homozygosis		(2) Mild reduction in oxidative growth and mitochondrial protein synthesis, moderate severe reduction in OCR<br>(3) Moderate reduced yeast growth
	Arg348Cys	Developmental delay, intellectual disability and/or microcephaly. In compound heterozygosis with His402Tyr		(2) Moderate severe reduction in oxidative growth, severe decrease in OCR. Moderate severe decrease in mitochondrial protein synthesis and protein levels<br>(3) Unable to support yeast growth

	Arg351Trp			(1) Unable to support yeast growth
	Asp377Asn	ARNSHI[6]. Homozygosis		(1) Mild reduced yeast growth<br>(2) Moderate reduced yeast growth<br>(3) Moderate reduced yeast growth
	His402Tyr	Developmental delay, intellectual disability and/or microcephaly. In compound heterozygosis with Arg348Cys		(2) Moderate severe reduction in oxidative growth, severe decrease in OCR and moderate severe decrease in mitochondrial protein synthesis<br>(3) Mild reduced yeast growth
	Glu427_Leu428insArg	Leukoencephalopathy. In compound heterozygosis with Leu596Phe		(1) Severe reduced yeast growth
	Pro499Leu	Developmental delay, intellectual disability and/or microcephaly. In compound heterozygosis with Phe291Val		(2) Thermosensitive mild reduction in oxidative growth, severe reduction in OCR and mitochondrial protein synthesis<br>(3) Unable to support yeast growth
	Arg505His	Leukoencephalopathy. In compound heterozygosis with Pro533Ser		(1) Severe reduced yeast growth
	Pro533Arg	Developmental delay, intellectual disability and/or microcephaly. In compound heterozygosis with Ala57Pro		(2) Absent oxidative growth, OCR and mitochondrial protein synthesis<br>(3) Unable to support yeast growth
	Pro533Ser	Leukoencephalopathy. In compound heterozygosis with Arg505His		(1) Mild reduced yeast growth
	Phe585Cys	Developmental delay, intellectual disability and/or microcephaly. In compound heterozygosis with Gln75Serfs*2		(2) Severe decrease in oxidative growth, OCR and mitochondrial protein synthesis<br>(3) Moderate reduced yeast growth

	Thr587Met	Leukoencephalopathy. In compound heterozygosis with Ile294Thr		(1) Mild reduced yeast growth
	Asn591Ile	Developmental delay, intellectual disability and/or microcephaly. Microcephaly. Homozygosis		(2) Absent oxidative growth, OCR and mitochondrial protein synthesis<br>(3) Moderate reduced yeast growth
	Leu596Phe	Leukoencephalopathy. In compound heterozygosis with Glu427_Leu428insArg		(1) Mild reduced yeast growth
LARS2	c. 1077delT	Perrault syndrome with POF[7] and sensorineural hearing loss. In compound heterozygosis with Thr522Asn	*nam2Δ* strain expressing human *LARS2* mutant alleles cloned in an integrating vector and harboring a mutation in *ADE2* gene, causing a red pigment production in presence of functional mitochondria	Non-functional protein
	Thr522Asn	Perrault syndrome with POF[7] and sensorineural hearing loss. Homozigosis		Reduced protein activity
	Thr629Met	Perrault syndrome with POF[7] and sensorineural hearing loss. In compound heterozygosis with c. 1077delT		Similar protein activity compared to wild type
RARS2	Trp241Arg	Pontocerebellar hypoplasia type 6. In compound heterozygosis with Gln12Arg	*msr1Δ* strain expressing *MSR1* mutant alleles cloned in a centromeric plasmid under CYC1 promoter or under its natural promoter	Similar oxidative growth compared to wild type
	Arg245Gln	Pontocerebellar hypoplasia type 6. In compound heterozygosis with Arg469His		Strong decrease in oxidative growth

	Arg469His	Pontocerebellar hypoplasia type 6. In compound heterozygosis with Arg245Gln		Absent oxidative growth and OCR
TARS2	Pro282Leu	Axial hypotonia and severe psychomotor delay associated with multiple mitochondrial respiratory chain defects. In compound heterozygosis with g.4255A_G; c.695 _ 3A_G	*ths1Δ* strain expressing *THS1* mutant alleles cloned in a centromeric plasmid under TEF promoter (1)<br><br>*ths1Δ* strain expressing human *TARS2* MTSΔ[3] mutant alleles cloned in a centromeric plasmid under TEF promoter (2)	(1) Severe reduction of protein levels<br>(2) Similar yeast cell growth compared to wild type
VARS2	Thr367Ile	Mitochondrial Encephalomyopathy. Homozygosis	*vas1Δ* strain expressing *VAS1* mutant alleles cloned in a centromeric plasmid under its natural promoter in presence of *cytVAS1* wild type allele	Mild reduced oxidative growth and OCR
	Thr647Met	Lactic acidosis with encephalomyopathy. In compound heterozygosis with Arg773Gln		Strong decrease in oxidative growth and OCR. Thermosensitive mild decrease in protein levels
	Arg773Gln	Lactic acidosis with encephalomyopathy. In compound heterozygosis with Thr647Met		Thermosensitive moderate decrease in oxidative growth and OCR. Thermosensitive moderate decrease in protein levels
WARS2	Val278Gly	Growth retardation and leukoencephalopathy. In compound heterozygosis with Lys313Met	*msw1Δ* strain expressing *MSW1* mutant alleles cloned	Absent oxidative growth and OCR

	Lys313Met	Growth retardation and leukoencephalopathy. In compound heterozygosis with Val278Gly	in a centromeric plasmid under its natural promoter	Thermosensitive moderate reduction in OCR
YARS2	Pro122Arg	MLASA2[8]. In compound heterozygosis with Leu208Arg	*msy1Δ* strain expressing *MSY1* mutant alleles cloned in a centromeric plasmid under its natural promoter	Absent oxidative growth and OCR
	Leu208Arg	MLASA2[8]. In compound heterozygosis with Pro122Arg		Moderate severe reduction in oxidative growth and OCR
	Asp311Glu	MLASA2[8]. Homozygosis		Strong reduction in oxidative growth, OCR and cytochromes content
	Cys369Tyr	MLASA[9]. In compound heterozygosis with Val383_Glu388dup		Thermosensitive strong reduction in oxidative growth. Moderate reduction in OCR
	Leu392Ser	MLASA[9]. Homozygosis		Moderate reduction in OCR

Supplementary Table 1. Human and yeast phenotype associated with mutations in ARS2. Numbers between "()" link yeast phenotype for each mutation modelled with the corresponding yeast model.

[1] Leukoencephalopathy with brainstem and spinal cord involvement and lactate elevation with early severe cerebral hypoplasia/atrophy

[2] Leukoencephalopathy with brainstem and spinal cord involvement and lactate elevation with whitematter abnormalities without long tract involvement

[3] Mitochondrial targeting signal

[4] Protein domain

[5] Spinal muscular atrophy

[6] Autosomal-recessive non syndromic hearing impairment

[7] Premature ovarian failure

[8] Myopathy, lactic acidosis, and sideroblastic anemia-2

[9] Myopathy, lactic acidosis, and sideroblastic anemia
